# Encoding and context-dependent control of reward consumption within the central nucleus of the amygdala

**DOI:** 10.1016/j.isci.2024.109652

**Published:** 2024-04-01

**Authors:** Kurt M. Fraser, Tabitha H. Kim, Matilde Castro, Céline Drieu, Yasmin Padovan-Hernandez, Bridget Chen, Fiona Pat, David J. Ottenheimer, Patricia H. Janak

**Affiliations:** 1 Department of Psychological & Brain Sciences, Krieger School of Arts & Sciences, Johns Hopkins University, Baltimore 21218, MD, USA; 2 Solomon H. Snyder Department of Neuroscience, Johns Hopkins School of Medicine, Baltimore 21205, MD, USA; 3 Johns Hopkins University Kavli Neuroscience Discovery Institute, Johns Hopkins School of Medicine, Baltimore 21205, MD, USA

**Keywords:** Biological sciences, Neuroscience, Behavioral neuroscience

## Abstract

Dysregulation of the central amygdala is thought to underlie aberrant choice in alcohol use disorder, but the role of central amygdala neural activity during reward choice and consumption is unclear. We recorded central amygdala neurons in male rats as they consumed alcohol or sucrose. We observed activity changes at the time of reward approach, as well as lick-entrained activity during ongoing consumption of both rewards. In choice scenarios where rats could drink sucrose, alcohol, or quinine-adulterated alcohol with or without central amygdala optogenetic stimulation, rats drank more of stimulation-paired options when the two bottles contained identical options. Given a choice among different options, central amygdala stimulation usually enhanced consumption of stimulation-paired rewards. However, optogenetic stimulation during consumption of the less-preferred option, alcohol, was unable to enhance alcohol intake while sucrose was available. These findings indicate that the central amygdala contributes to refining motivated pursuit toward the preferred available option.

## Highlights

• Central amygdala neurons are modulated by the consumption of alcohol and sucrose
• Central amygdala neurons exhibit significant lick modulation during consumption
• Excitation of central amygdala promotes consumption of the most valued reward
• Central amygdala inhibition decreases consumption only if the rewards are equal

## Graphical abstract

**Figure:**
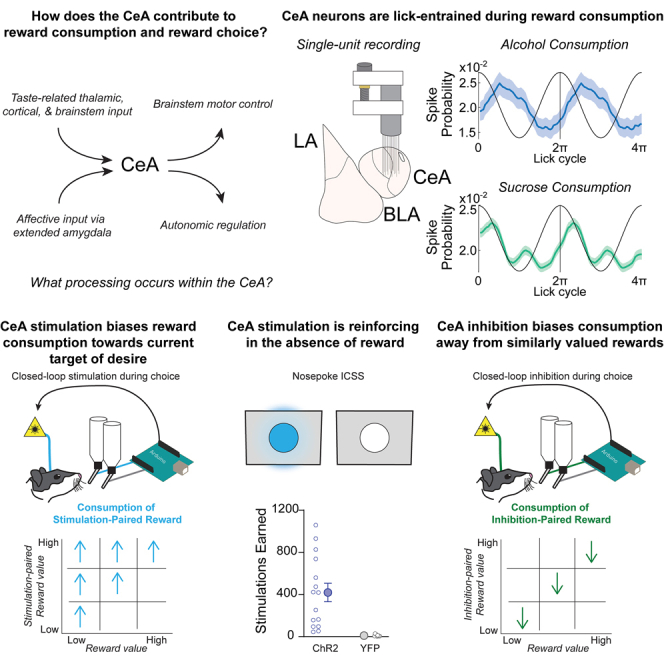

## Introduction

Selecting the best available option requires an assessment of the rewards currently available and a comparison of their relative values to appropriately direct motivated behavior. In substance use disorders, motivation becomes focused onto the pursuit of drug rewards despite the availability of other, perhaps more optimal, options.^1^^,^^2^^,^^3^ The central amygdala (CeA) has been implicated in these processes as lesions or inactivation of this nucleus reduce drug self-administration and optogenetic stimulation of the CeA can promote choice of a stimulation-paired option over an otherwise equivalent reward.^4^^,^^5^^,^^6^^,^^7^^,^^8^^,^^9^^,^^10^^,^^11^^,^^12^^,^^13^^,^^14^ However, these investigations have primarily been conducted with only one outcome type available, be it drug or natural rewards, and preclude an assessment of the contributions of the CeA to the dynamic process of choosing which outcome to pursue.^15^^,^^16^

The CeA is a candidate for reward valuation, including ingested rewards, as it receives privileged input from taste processing brainstem, thalamic, and cortical regions.^17^^,^^18^^,^^19^^,^^20^ Previous work identified robust responses of CeA neurons to ingested outcomes^21^^,^^22^^,^^23^^,^^24^^,^^25^^,^^26^ and found pharmacological and optogenetic stimulation of some CeA cell populations increases intake of food or liquids^21^^,^^27^^,^^28^ further supporting a role for the CeA in a valuation process. While this work has begun to elucidate the means by which CeA neurons contribute to reward consumption more generally,^21^^,^^22^ less is known about how neural activity within the CeA is related to alcohol consumption and choice *in vivo*. Oral alcohol not only shares sensory properties with ingested outcomes but also has pharmacological properties that themselves profoundly impact CeA circuitry. Acute and, especially, chronic alcohol exposure alters CeA gene expression and synaptic physiology.^29^^,^^30^^,^^31^^,^^32^^,^^33^ In addition, learning-dependent changes as a result of associating the taste of alcohol with its pharmacological effects may impact CeA function.^32^^,^^34^ Of note, the taste of alcohol selectively activates mTORC1 signaling in the CeA and prelimbic and orbitofrontal cortices of alcohol-experienced rats and this activation of the CeA is necessary for the taste of alcohol to motivate alcohol-seeking.^34^ Recently, Torruella-Suárez and colleagues demonstrated that manipulation of CeA neurotensin neurons in mice alters both alcohol and sucrose intake, further implicating this area in the regulation of alcohol intake.^11^

Here we examined the contributions of CeA neuronal activity to alcohol consumption in rats with a prior history of intake of alcohol at high levels to better understand how alcohol consumption may engage this region. In addition, we tested the contributions of CeA activity to reward choice, using optogenetic manipulation of the CeA time-locked to consumption to alter the choice between pursuit of alcohol over other options. We reveal robust neural responses to alcohol and natural reward consumption in the CeA and find that optogenetic increases or decreases of CeA neural activity can increase or decrease reward consumption, respectively. Of note, while the consumption of rewards modulates CeA neural activity, the impact of CeA activation or inhibition is constrained by subjects’ preference between reward and drug options currently available in the environment. Collectively, our findings suggest that the CeA acts as a motivational filter to focus reward and alcohol pursuit in a context-dependent manner.

## Results

### Encoding of alcohol consumption within the central nucleus of the amygdala

**Figure 1:**
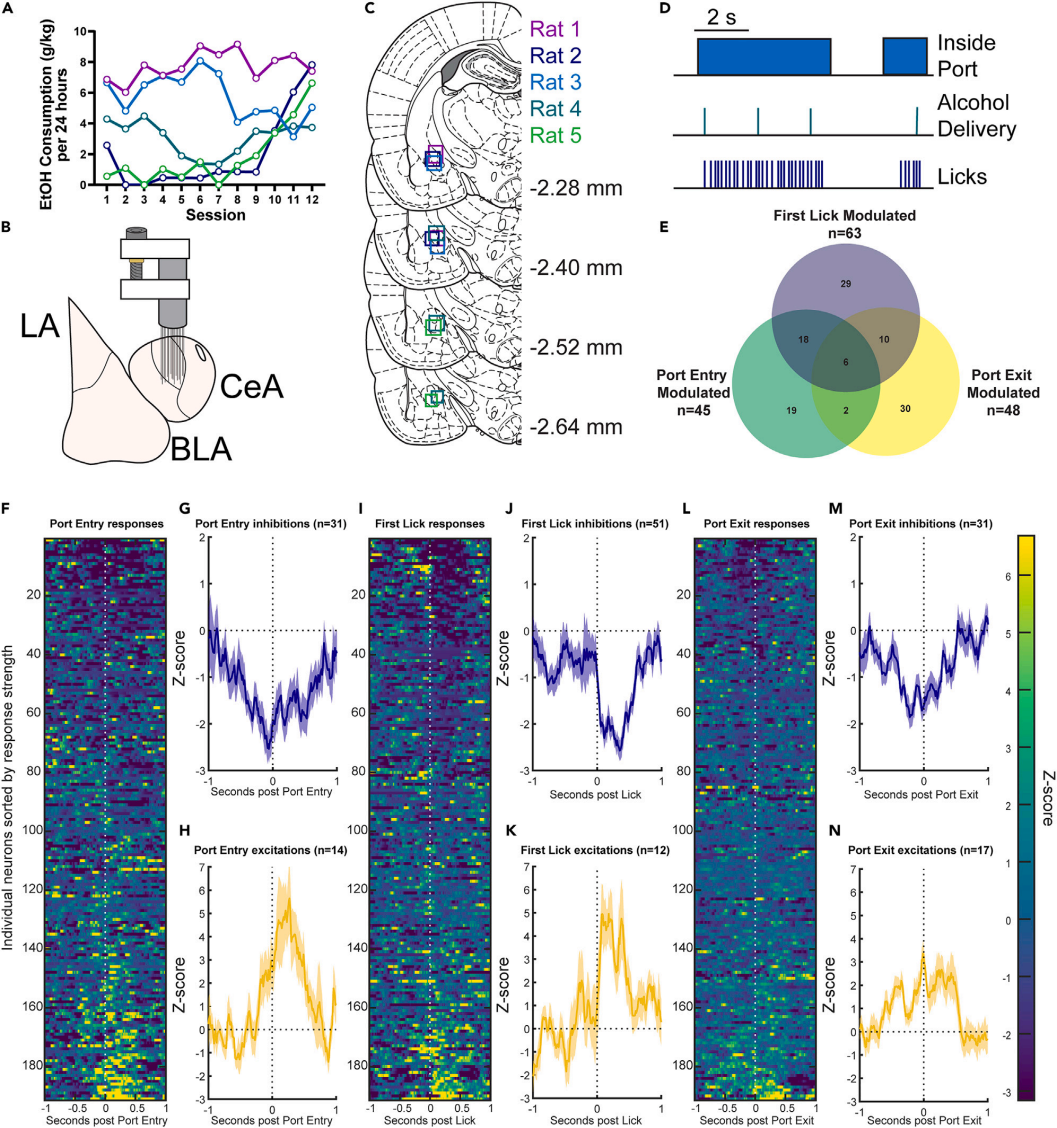
Identifying correlates of alcohol consumption in the central nucleus of the amygdala (A) Consumption of alcohol during a 4-week every other day, 24-h availability to 15% alcohol on an intermittent access schedule expressed in g alcohol consumed per kg body weight. (B) Schematic of recording strategy of 16 50-μm diameter tungsten wires in a drive targeted to the central amygdala. (C) Recreation of recording sites from each of the five rats. (D) Overview of task design. Sessions started with a prime of alcohol delivery in the port, and every cumulative 2 s spent in the port thereafter triggered a new delivery of alcohol. (E) Proportion of neurons significantly excited or inhibited by task-relevant events. (F) Heatmap of z-scored responses for each neuron recorded sorted by the strength of excitation to port entry. (G) Average z-scored response of all neurons that were identified as being significantly inhibited around port entry. (H) Average z-scored response of all neurons that were identified as significantly excited around port entry. (I–K) Same as F–H but for the first lick post port entry. (L–N) Same as F–H but for port exit. Heatmaps are sorted individually for each event. Traces indicate mean z-scored response with overlaid bands indicating ±1 standard error of the mean.

To evaluate potential correlates of alcohol consumption we pre-exposed rats in their homecage to 15% alcohol on an intermittent access schedule 3 days a week for 4–5 weeks.^35^^,^^36^ This allowed rats to become familiar with the taste of alcohol and to associate alcohol consumption with its pharmacological effects, and, as well, to develop a propensity to consume relatively high levels of alcohol (Figure 1A). Following initial homecage pre-exposure, rats were implanted with drivable bundles of electrodes targeted to the CeA (Figures 1B and 1C) and neural activity was measured in freely moving rats during alcohol consumption from a reward port situated in a standard behavioral chamber. In these self-administration sessions, rats could enter the port to drink alcohol from a small receptacle that was primed with alcohol at session start. Once rats entered the port, a cumulative presence of 2 s activated the pump to deliver 0.1 mL of alcohol, a volume chosen to approximate the volume of alcohol typically consumed within 2 s (Figure 1D). Thus we approximated continuous liquid delivery with no breaks when subjects chose to drink. Once electrophysiological recording began, there was no longer alcohol available in the homecage, so rats had to earn alcohol in these 40 min, every-other-day recording sessions. We recorded 208 well-isolated single-units in 5 rats from the CeA during the consumption of alcohol with an average firing rate of 3.34 ± 0.198 (mean ± SD) Hz. During these sessions, rats made on average per session 29.54 ± 2.76 port entries, 332.3 ± 43.78 licks, and drank an average of 0.75 ± 0.07 g/kg of alcohol. This level of alcohol consumption in this 40 min time frame produces blood alcohol contents of approximately 17.37 ± 4.24 mg/dL (data from a cohort of rats solely tested for alcohol consumption; *n* = 17). We identified diverse neural responses to consumption-relevant behaviors (Figure 1E), primarily decreases in firing at approach (Figures 1F–1H) to the alcohol and exit (Figures 1L–1N) from the alcohol-containing port. Close inspection of the responses of single neurons that were significantly modulated by the first lick (Figures 1I–1K) suggested that these neurons may be rhythmically modulated by the protrusion and retraction of the tongue, leading us to conduct further analyses to characterize CeA neural correlates of alcohol consumption.

**Figure 2:**
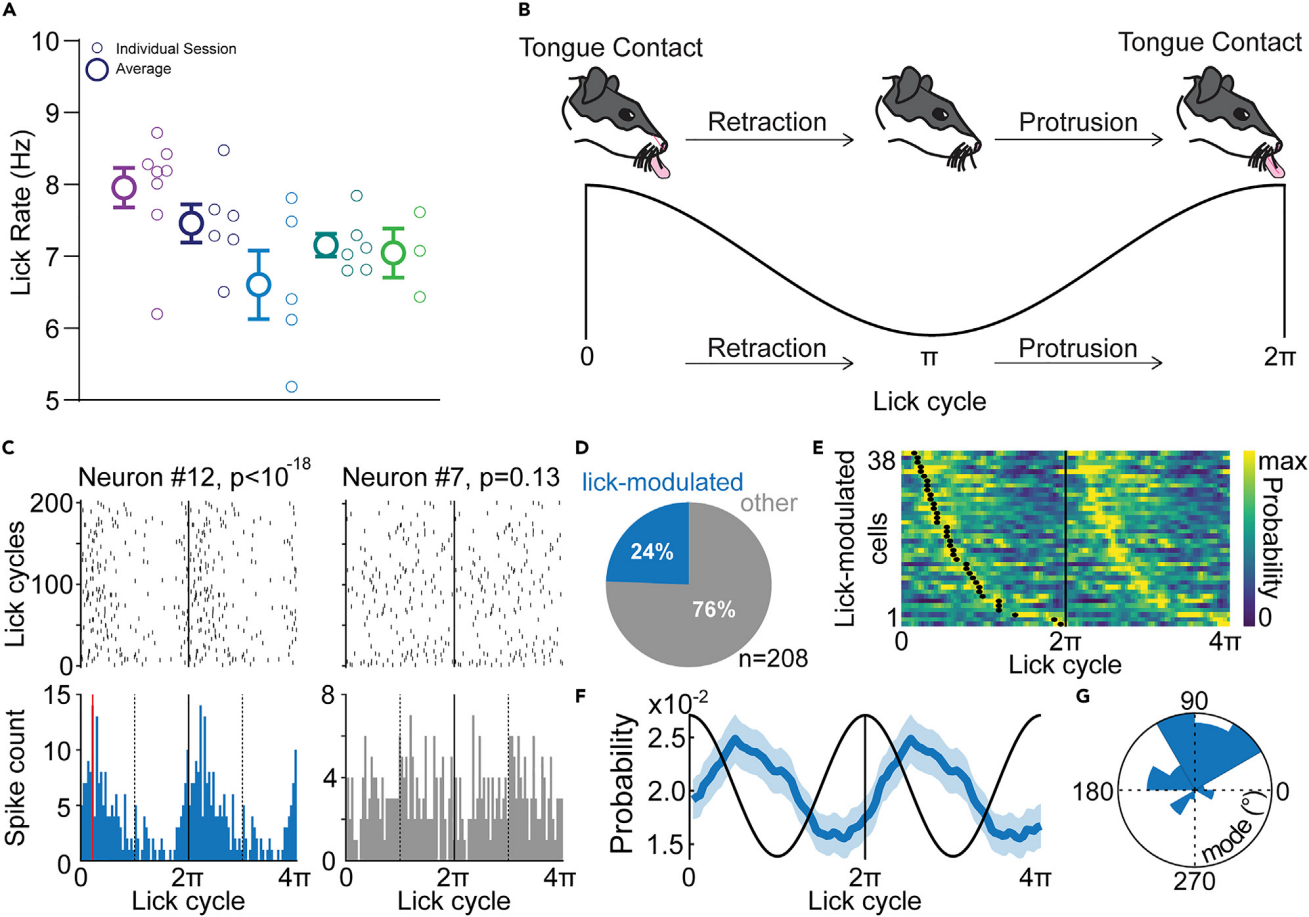
Central amygdala neurons are modulated by licks during the consumption of alcohol (A) Average lick rates during the consumption of 10% alcohol for each rat during recording session. Smaller symbols indicate lick rate for each individual session. (B) Schematic representation of an individual lick cycle and its analogous sinusoidal rhythm. Times 0 and 2π represent contact with the liquid reward. (C) Spike rasters (top) and histograms (bottom) during lick cycles for two example neurons recorded in the same session. A lick cycle is defined as the time between two consecutive contacts with the fluid delivery port (see STAR methods). The *p*-value of Rayleigh test is indicated. (D) Proportion of neurons significantly modulated by licks (Rayleigh’s test with *p*-value <0.01). (E) Heatmap of spike probability during lick cycles of lick-modulated neurons. Black dots indicate the preferred firing phases (i.e., modes). (F) Average spike probability of lick-modulated neurons across lick cycles (mean ± SEM). (G) Circular histogram of the preferred firing phases (V test against 90°, *n* = 38 lick-modulated neurons, V_38_ = 19.92, *p* < 10^−5^).

To better relate CeA activity to the ongoing consumption of alcohol, we first analyzed the average lick rate for each recording session. Rat licking is highly stereotyped and typically averages 7 licks per second but can vary within the 5–10 Hz frequency range.^37^^,^^38^ We observed similar average lick rates within this frequency band during alcohol consumption, averaging around 7 Hz across sessions and between rats (Figure 2A). Next, we assessed whether the firing of each of the 208 CeA neurons was modulated by rhythmic licking. To do so, we computed the firing phases of each neuron relative to the lick cycle (phase 0 represents the contact of the tongue with the fluid delivery port; Figure 2B) and tested whether these firing phases were uniformly distributed across the lick cycle. Neurons with *p*-values below 0.01 were considered lick-modulated (Rayleigh test for non-uniformity; Figure 2C). We observed significant lick-modulation in the CeA as rats consumed ethanol, with 24% of the neuronal population exhibiting significant lick-modulation (Figure 2D). At the population level, the large majority of lick-modulated neurons maximally fired early in the lick cycle, right after contact with alcohol, during the retraction of the tongue into the mouth (Figures 2E and 2F). Consistent with a previous description of lick coherent firing of amygdala neurons,^39^ the distribution of the preferred firing phases showed that ∼84% (32/38) of the lick-modulated neurons preferentially fired between 0 and π, with an overall mean direction of π/2 (Figure 2G). This suggests that, in addition to encoding the approach to alcohol, CeA neurons are phase locked to the lick rhythm during alcohol consumption and preferentially fired during the retraction of the tongue into the mouth right after the retrieval of alcohol from the environment.

We also recorded neurons in the CeA during the consumption of a concentration of sucrose isocaloric to 10% ethanol. We observed similar overall patterns of activity at the time of sucrose approach (Figure S1) and lick-modulation in the CeA during sucrose consumption (Figure S2) suggesting common codes for consumption of rewards in the CeA. Interestingly, we observed more lick-entrainment in the CeA of rats consuming sucrose than for the consumption of ethanol (Figure S2G). However, there was no difference in the preferred phase of the lick cycle for lick-modulated CeA neurons during alcohol and sucrose consumption (Figure S2H). This observation suggests that the degree of lick-modulation in the CeA neuronal population is influenced by reward value. Together, these data indicate that the CeA is modulated by the pursuit of reward and is strongly engaged during the ongoing consumption of both natural and drug rewards.

### Optogenetic excitation of the central amygdala biases reward choice but is filtered by preference

**Figure 3:**
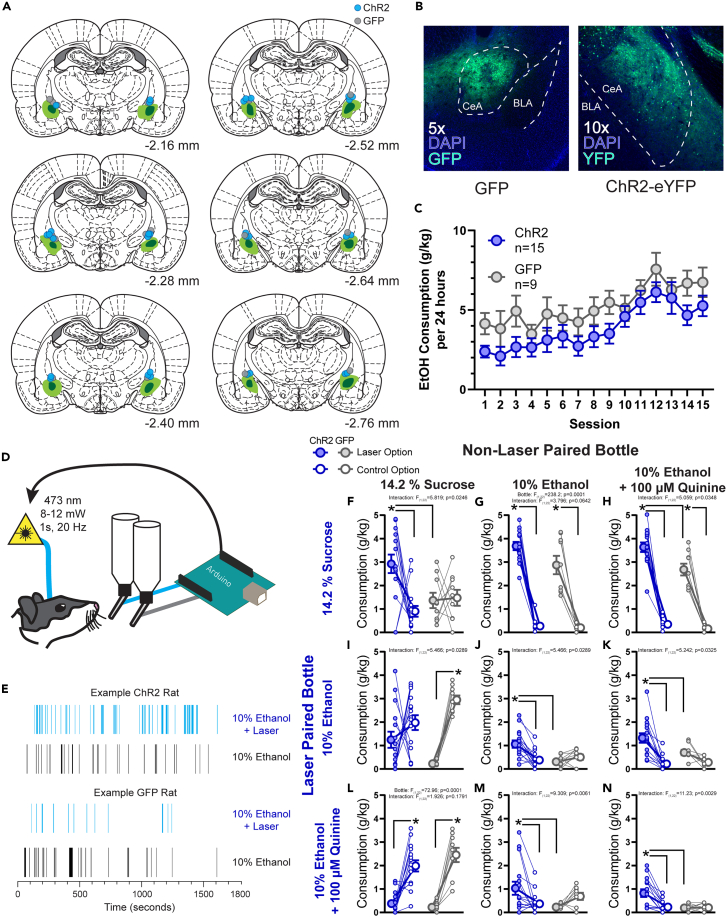
Context-dependent enhancement of reward consumption resulting from closed-loop optogenetic stimulation of the central amygdala (A) Reconstruction of the maximal (light green) and minimal (dark green) viral expression of either hsyn-ChR2-eYFP or hsyn-GFP within the central amygdala. Blue dots indicate fiber tips for rats with ChR2 and gray dots indicate fiber tips for rats with GFP. (B) Example images of expression of GFP and ChR2-eYFP within the central amygdala (green) and nuclear staining with DAPI in blue. (C) Homecage alcohol consumption (in g alcohol per kg body weight) on an every-other day, 24 h intermittent access schedule to 15% alcohol. Consumption in the homecage did not differ between rats with ChR2 or GFP expression in the central amygdala (F_1,22_ = 2.658, *p* = 0.1172). (D) Rats were allowed to freely direct their consumption between two bottles containing a variety of liquid rewards. Licks were recorded on each bottle by an Arduino and in turn the first lick each second on one of the bottles would result in a 1 s, 20 Hz train of blue light delivered bilaterally to the central amygdala. (E) Example lick rasters from a session in which both bottles contained 10% alcohol for a representative ChR2 rat and a representative GFP rat. (F–N) Consumption of each solution in g consumed per kg body weight. Graphs are organized with the most valued option at the top and leftmost position and the least valued option at the bottom and rightmost position. Comparisons between bottles containing the same offer tile the diagonal, bottles above diagonal are tests in which the more valued option was blue-light paired and tests below the diagonal are when blue-light was paired with the less valued option. Blue symbols represent ChR2 rats and gray symbols indicate GFP rats. Filled symbols indicate the bottle that resulted in blue light delivery, open symbols the other bottle that did not trigger any light delivery. Large symbols indicate group means ±1 standard error of the mean and small symbols represent individual rats. ∗*p* < 0.05 for post hoc comparisons made only when a significant main effect of bottle or an interaction between virus group and bottle were observed.

We observed correlates of consumption and motivation to pursue alcohol in the CeA and sought to understand the functional consequences of this activity. To do this, we expressed either the excitatory, blue-light activated opsin ChR2 or a control fluorescent protein GFP in the CeA of rats (Figures 3A and 3B). After surgery for virus infusion and optical fiber placement, rats became familiar with the taste and pharmacological properties of alcohol in their homecage by allowing rats 24 h of access to 15% alcohol 3 days a week for 5 weeks (Figure 3C). After this homecage alcohol pre-exposure, optogenetic manipulation during choice tests commenced. We gave rats 30-min access to pairs of solutions, comprised of different combinations of 10% alcohol, isocaloric 14.2% sucrose, and 10% alcohol adulterated with 100 μM quinine, an outcome commonly used to test for compulsive consumption of alcohol,^40^^,^^41^^,^^42^ in a modified homecage. For each test session, licks were recorded from each of the two bottles. Licks made on one of the bottles triggered deliveries of a 1 s, 20 Hz train of 8–12 mW blue light bilaterally into the CeA to drive stimulation in a closed-loop manner tied to ongoing consumption (Figure 3D). In effect, during a given drinking bout on the laser-paired bottle, a subject would have near constant optogenetic stimulation.

We found that rats expressing ChR2 in the CeA would consume more of the laser-paired option if the other non-laser paired option was identical (Figure 3E for example licking behavior at test). This was true for sucrose (Figure 3F), alcohol (Figure 3J), and quinine-adulterated alcohol (Figure 3N). We examined the microstructure of consumption to determine the psychological mechanisms underlying this effect, focusing on clusters of licks (drinking bouts) and the number of licks within a cluster. Cluster number is typically taken to reflect motivation for a particular substance while lick number per cluster is correlated with palatability.^37^^,^^43^^,^^44^^,^^45^ In GFP control rats, mean cluster number and cluster length were correlated with reward value/palatability with sucrose>alcohol>quinine-adulterated alcohol, as expected. We observed that stimulation primarily increased the number of drinking bouts made to the laser-paired option, but rarely increased the average number of licks within each cluster, as compared to GFP controls, with the interesting exception of quinine-adulterated alcohol (Figure S3). If the stimulation were directly activating licking motor pattern generators, a consistent increase in the length of drinking clusters might be expected. Instead, this pattern of increased clusters, or number of drinking bouts, suggests that CeA stimulation enhanced the motivation to pursue and consume the laser-paired option but had less effect on the perceived palatability of the option.^37^^,^^43^^,^^45^

Next, we asked whether CeA stimulation would alter the choice rats made between two different options. Interestingly, we found that if the laser-paired option was preferred by the rats, then stimulation enhanced consumption of the preferred reward even further above that observed in control rats. For example, optogenetic stimulation enhanced intake of sucrose and of alcohol when each of these was pitted against quinine-adulterated alcohol (sucrose over alcohol-quinine Figure 3H; alcohol over alcohol-quinine Figure 3K). While the interaction between bottle and group did not reach significance for laser-paired sucrose consumption with alcohol as the other option (Figure 3G), this is potentially due to a ceiling effect of consumption in this time-limited test. Nonetheless, stimulation paired with reward ingestion in all of these cases significantly increased motivation to consume the laser-paired option as evidenced by increased drinking bouts, but did not appear to alter the hedonic value (i.e., palatability) of the laser option, as can be seen by comparing the number of licks per cluster in ChR2 subjects vs. GFP controls (Figure S3).

In contrast, when CeA stimulation was paired with the non-preferred option, and sucrose was the other option available in the choice, stimulation did not reliably increase consumption of the laser-paired alcohol options. This was especially apparent in the case of alcohol-quinine vs. sucrose, where stimulation had no impact on intake (Figure 3L). Interestingly, stimulation also failed to increase alcohol intake over sucrose when analyzing intake (Figure 3I), but stimulation did increase the mean number of lick clusters for alcohol vs. sucrose (Figure S3N), suggesting a limited ability of CeA stimulation paired with alcohol consumption to promote choice of that option over other possible rewards. Elevated motivation by some ChR2 rats to consume alcohol when sucrose was the non-stimulated option was not related to overall measures of propensity to drink alcohol, as the correlation between alcohol intake on the laser-paired bottle and their final level of homecage alcohol consumption was not significant (r = −0.4534, *p* = 0.0896).

Surprisingly, when alcohol was the control offer and quinine-adulterated alcohol was stimulation-paired, ChR2 rats drank more quinine-adulterated alcohol (Figure 3M) and increased the number of lick clusters (Figure S3S), perhaps representative of reduced sensitivity to punishment in some individuals^40^ upon CeA activation. Of note, there was no correlation between alcohol-quinine intake when it was laser-paired and alcohol was available and the final homecage alcohol drinking session (r = −0.3988, *p* = 0.1409) for ChR2 rats.

This pattern of findings suggests that the CeA directs motivation to the most preferred offer but that underlying reward preferences affecting choice may be independent of the CeA. However, sucrose is highly valued and more palatable than alcohol, a frequently less-preferred outcome in non-dependent rats, and we wondered if this pattern of results could generalize to comparisons among more similarly valued non-drug outcomes. To better isolate the possibility of selective enhancement of motivation to preferred outcomes, we offered rats the choice between sucrose and maltodextrin, two isocaloric sweet rewards that are consumed at equivalent levels when presented in isolation but differ in that rats prefer to consume sucrose when given a choice.^38^^,^^46^^,^^47^^,^^48^ When both bottles contained maltodextrin, ChR2 rats consumed more of the maltodextrin leading to CeA stimulation (Figure S4). When CeA stimulation was paired with sucrose consumption while maltodextrin was available, CeA stimulation enhanced sucrose consumption above that observed in control rats just as we observed with choice between dispartely valued laser-paired sucrose and alcohol. Of note, when CeA stimulation was paired with maltodextrin consumption and the more preferred sucrose was the other option, ChR2 rats enhanced maltodextrin consumption, reversing preference between the two rewards (Figure S4). Thus it is possible for CeA stimulation to amplify reward consumption of a less preferred option if the value of available options are similar.

These findings collectively indicate that enhancing CeA neural activity can strongly bias reward choice resulting in increased consumption of the laser paired option, even for drugs of abuse, especially if that option is of greater or similar value than other offers. In contrast, when rats chose between alcohol (less preferred) and sucrose (more preferred), CeA stimulation does not reliably increase alcohol intake above sucrose intake.

### Optogenetic stimulation of the central amygdala is reinforcing

**Figure 4:**
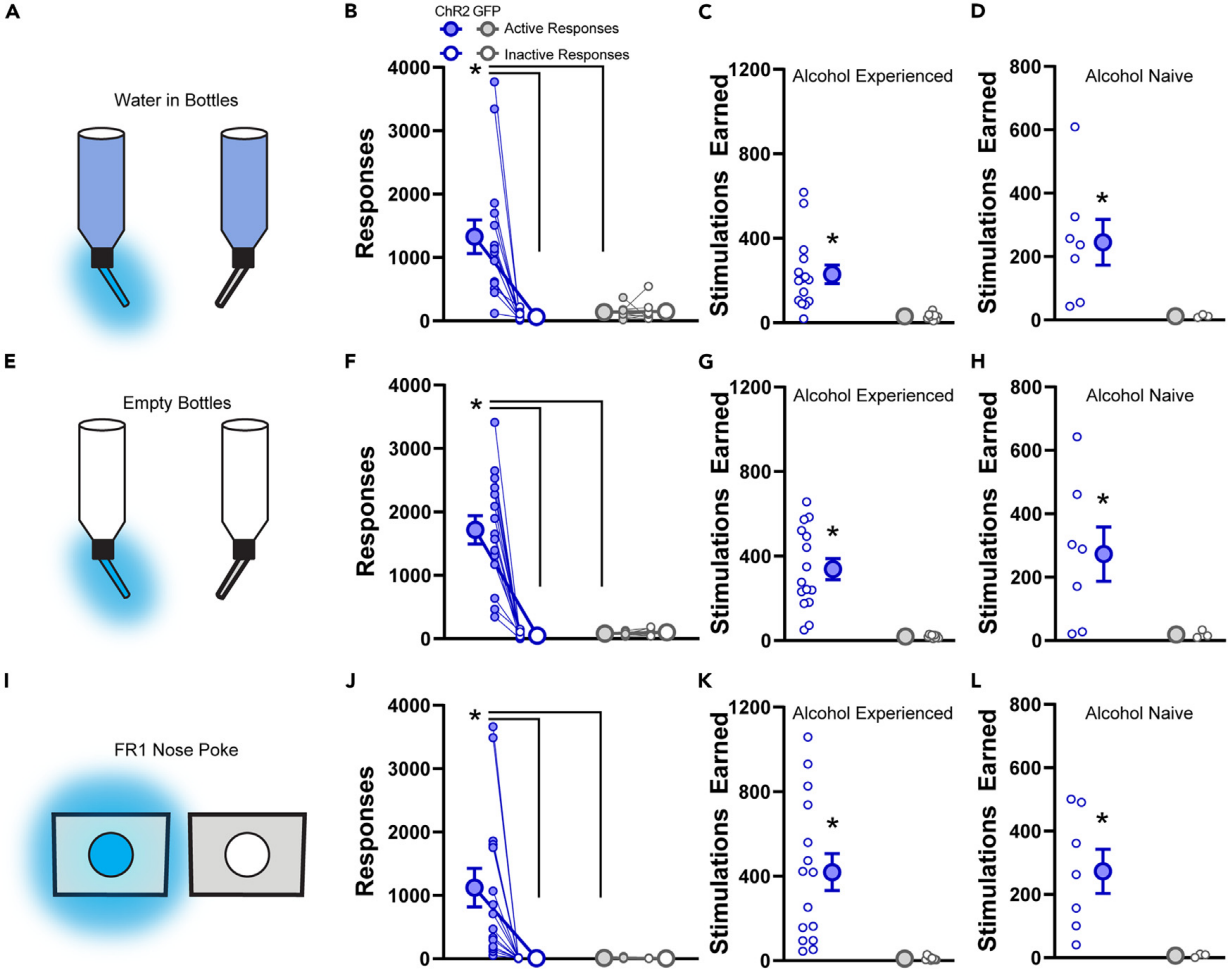
Optogenetic self-stimulation of the central amygdala is reinforcing regardless of alcohol experience (A) Rats were allowed 30 min of access to water-containing bottles in the modified homecage and the first lick to one bottle resulted in a 1s, 20 Hz train of blue light delivered bilaterally to the central amygdala. (B) Rats with ChR2 in the central amygdala made significantly more licks to the stimulation-paired bottle than the control bottle and also more than GFP rats. (C) Total stimulations earned during the session. (D) Total stimulations earned for a subset of rats tested prior to any alcohol drinking experience. (E–H) same as A–D but for a test in which both bottles were empty. ChR2 rats made significantly more licks to the stimulation paired bottle and earned significantly more stimulations than GFP rats. (I–L) Same as A–D but instead of bottles, rats were placed in operant conditioning chambers and allowed to nose poke freely where the first poke each second in port resulted in a 1s, 20 Hz train of blue light delivered bilaterally into the central amygdala. Filled symbols indicate the bottle that resulted in blue light delivery, open symbols the other bottle that did not trigger any light delivery. Large symbols indicate group means ±1 standard error of the mean and small symbols represent individual rats. ∗*p* < 0.05 for post hoc comparisons made only when a significant main effect of bottle or an interaction between virus and bottle were observed.

CeA stimulation generally enhanced consumption of the most valuable available reward, highlighting a possibility for an innate reinforcing property of CeA stimulation that could promote responding absent physical reward. Evidence for such a primary reinforcement signal in the CeA has been mixed.^9^^,^^10^^,^^11^^,^^12^^,^^13^^,^^14^^,^^49^^,^^50^ We first gave rats the option to drink water that was paired with CeA stimulation. As before, stimulation increased the consumption of laser-paired water, which was surprising given rats were not water-restricted (Figures 4A–4C; response: F_1,22_ = 12.56, *p* = 0.0018; group: F_1,22_ = 10.24, *p* = 0.0041; interaction: F_1,22_ = 12.98, *p* = 0.0016; stimulations: t_14_ = 4.491, *p* = 0.0005). We then asked if rats would respond without reward available to earn CeA stimulation by offering empty bottles. We observed rats would lick on the empty bottle to earn CeA activation (Figures 4E–4G; response: F_1,22_ = 30.45, *p* < 0.0001; group: F_1,22_ = 30.59, *p* < 0.0001; interaction: F_1,22_ = 32.05, *p* < 0.0001; stimulations: t_14_ = 6.377, *p* < 0.0001). We then wanted to rule out potential extinction effects contributing to this behavior as the rats had extensive experience in drinking from bottles in the testing apparatus. We placed rats into a novel operant behavioral chamber with two nosepoke ports available. Responses in one nosepoke led to CeA stimulation. Rats acquired this novel response and worked to earn CeA stimulation (Figures 4I–4K; response: F_1,22_ = 8.019, *p* = 0.0097; group: F_1,22_ = 7.92, *p* = 0.0101; interaction: F_1,22_ = 7.807, *p* = 0.0106; stimulations: t_14_ = 4.674, *p* = 0.0004). A subset of rats (*n* = 7 ChR2, *n* = 3 GFP) were tested for intracranial self-stimulation prior to homecage ethanol drinking to assess whether the consistent self-stimulation we observed could result from ethanol-induced plasticity. However, even alcohol-naive rats exhibited self-stimulation of the CeA (Figures 4D, 4H, and 4L; water: t_6_ = 3.23, *p* = 0.0178; empty:t_6_ = 2.959, *p* = 0.0249; nosepoke: t_6_ = 3.809, *p* = 0.0088). These data indicate there is a reinforcing property of CeA stimulation itself but, in the case of external reward availability, this reinforcing property is filtered by preference among the available rewards.

### Optogenetic inhibition of the central amygdala reduces reward intake under limited circumstances

**Figure 5:**
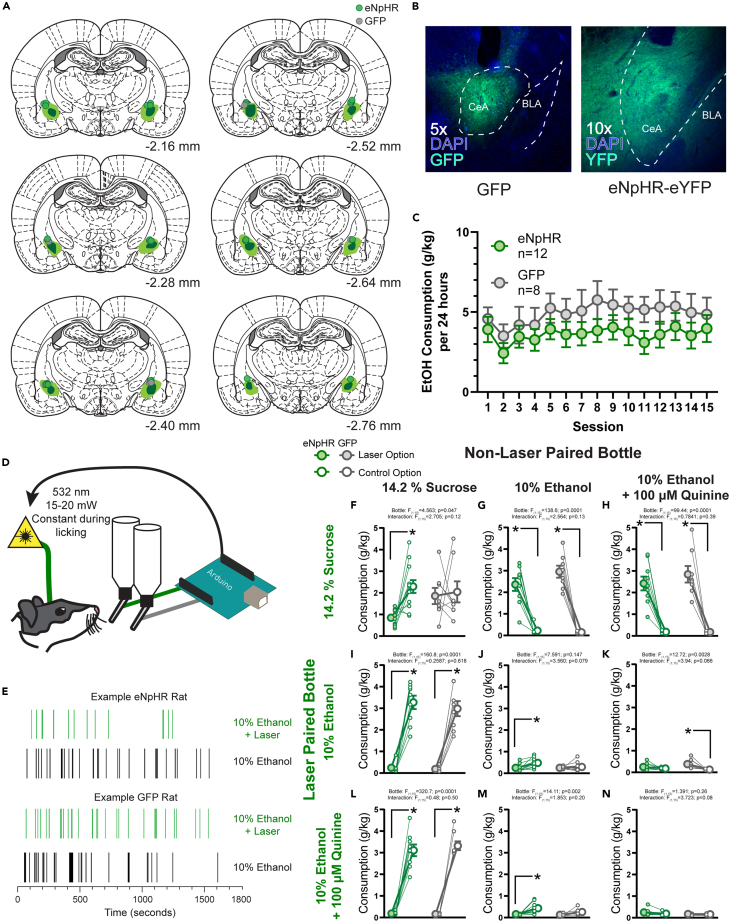
Context-dependent suppression of reward consumption resulting from closed-loop optogenetic inhibition of the central amygdala (A) Reconstruction of the maximal (light green) and minimal (dark green) viral expression of either hsyn-eNpHR-eYFP or hsyn-GFP within the central amygdala. Green dots indicate fiber tips for rats with eNpHR and gray dots indicate fiber tips for rats with GFP. (B) Example images of expression of GFP and eNpHR-eYFP within the central amygdala (green) and nuclear staining with DAPI in blue. (C) Homecage alcohol consumption (in g alcohol per kg body weight) on an every-other day, 24 h intermittent access schedule to 15% alcohol. Consumption in the homecage did not differ between rats with eNpHR or GFP expression in the central amygdala (F_1,18_ = 1.695, *p* = 0.2093). (D) Rats were allowed to freely direct their consumption between two bottles containing a variety of liquid rewards. Licks were recorded on each bottle by an Arduino and in turn the first lick on one of the bottles would result in a constant train of green light delivered bilaterally to the central amygdala. (E) Example lick rasters from a session in which both bottles contained 10% alcohol for a representative eNpHR rat and a representative GFP rat. (F–N) Consumption of each solution in g consumed per kg body weight. Graphs are organized with the most valued option at the top and leftmost position and the least valued option at the bottom and rightmost position. Comparisons between bottles containing the same offer tile the diagonal, bottles above diagonal are tests in which the more valued option was green-light paired and tests below the diagonal are when green-light was paired with the less valued option. Green symbols represent eNpHR rats and gray symbols indicate GFP rats. Filled symbols indicate the bottle that resulted in green light delivery, open symbols the other bottle that did not trigger any light delivery. Large symbols indicate group means ±1 standard error of the mean and small symbols represent individual rats. ∗*p* < 0.05 for post hoc comparisons made only when a significant main effect of bottle or an interaction between virus group and bottle were observed.

We observed that stimulation of the CeA can increase the motivation to pursue and consume rewards. We were then interested in examining the effects of inhibition of the CeA. To do this, we expressed either the inhibitory, green- and yellow-light activated opsin halorhodopsin (eNpHR) or a control fluorescent protein GFP in the CeA of rats (Figures 5A and 5B), and trained rats as above to choose from bottle pairs presented in 30 min tests; for each test, licks were recorded from each of the two bottles and licks made on one of the bottles were associated with the delivery of a constant train of 15–20 mW green light bilaterally into the CeA until no lick was detected for 1 s (Figure 5D).

Overall, the effects of optogenetic inhibition of the CeA on reward consumption were much less robust than those of optogenetic excitation. When examining choices among sucrose, alcohol, and alcohol-quinine, almost none of the two-way ANOVAs comparing virus group and bottle revealed significant interactions. We considered that reductions in already relatively low levels of alcohol intake would be difficult to detect due to floor effects, and therefore chose to conduct post hoc comparisons within each virus group across the two bottles to evaluate our *a priori* hypotheses based on our strong findings with optogenetic stimulation.

We found that rats with eNpHR in the CeA consumed less of the laser-paired option if the other non-laser paired option was identical (Figure 5E for example licking behavior at test). This was true for sucrose (Figure 5F) and alcohol (Figure 5J). Baseline consumption of quinine-adulterated alcohol was already low so there was no effect of inhibition (Figure 5N). We examined the microstructure of consumption to determine the psychological mechanisms underlying this effect. In cases where a significant decrease in intake was observed, we did not find a significant decrease in the number of clusters of licks made to the laser paired option but did find a decrease in the average number of licks within each cluster for sucrose but not alcohol (Figure S5). This could indicate that inhibition of CeA impacts palatability. However, given that consumption was low, this decrease may be a result of floor effects of overall consumption from the limited 30-min sessions.

We then asked whether CeA inhibition would alter the choice rats made between two different options. We found that if the laser-paired option was preferred by the rats, (e.g., sucrose over alcohol, alcohol versus quinine-adulterated alcohol) inhibition could not reduce consumption of the preferred reward below that observed in control rats (sucrose over alcohol Figure 5G; sucrose over alcohol-quinine Figure 5H; alcohol over alcohol-quinine Figure 5K). In addition, when CeA inhibition was paired with the non-preferred option, the laser had no effect on consumption (alcohol vs. sucrose Figure 5I; alcohol-quinine vs. sucrose Figure 5L; alcohol-quinine vs. alcohol Figure 5M).

Once again, we offered rats the choice between sucrose and maltodextrin to compare the ability of optogenetic inhibition of the CeA to alter choice between two similarly valued rewards. When both bottles contained maltodextrin, there was a borderline interaction between bottle and group, accounted for by lower consumption by eNpHR rats of maltodextrin paired with CeA inhibition (Figure S6B). When CeA inhibition was paired with sucrose consumption when maltodextrin was available, CeA inhibition did not reduce sucrose consumption (Figure S6A). When CeA inhibition was paired with maltodextrin consumption and the more preferred sucrose was the other option, both eNpHR and GFP rats consumed more sucrose (Figure S6C), and consumption of maltodextrin was not significantly decreased below control levels. Unlike stimulation of the CeA, inhibition of the CeA only reduced reward preference in closed-choice scenarios. Taken together, CeA inhibition had subtle effects on reward intake, reducing consumption of the laser-paired option when the other option was of equal value, but it did not reverse preference between disparately valued rewards.

## Discussion

Here we describe encoding of alcohol consumption in the central amygdala and provide a demonstration of conditions in which central amygdala stimulation and inhibition can alter alcohol and other reward preference. We report a phasic signal in central amygdala neurons during licking for both drug and natural rewards that is entrained to the lick cycle. In addition, when optogenetic manipulation of the central amygdala is time-locked to the consumption of rewards we reveal a context-dependent ability of stimulation and inhibition to alter consumption. While optogenetic stimulation of the central amygdala generally increased intake of a given reward, in a context where alcohol and sucrose are the two available choices, only when the most preferred currently available reward, in this case, sucrose is paired with stimulation does the central amygdala contribute to prolonged pursuit and consumption. Further, optogenetic inhibition of the central amygdala overall had a weaker impact, decreasing consumption when options in the environment were the same (both bottles containing sucrose or alcohol). Together, these findings provide evidence that the central amygdala is a critical node in decision-making circuitry that integrates value-related information about available rewards to filter and refine motivation.

The CeA is historically known as critical for the scaling and expression of fear-related responses,^51^ with the interaction between the CeA and primary taste centers in the brain receiving relatively less attention. The taste-related afferents into the CeA may allow for the integration of the hedonic properties of reward with relevant state- and history-dependent representations within the CeA. Indeed, afferents from the parabrachial nucleus and insular cortex to the CeA are essential for the avoidance of a previously appetitive tastant made aversive through pairing with gastrointestinal distress.^52^^,^^53^ CeA inputs from the insula, either direct or indirect via the basolateral amygdala, have been proposed to mediate assignment of taste value.^27^ The CeA could then integrate this taste information into relevant cell-type and projection-specific circuits to select the appropriate consummatory action and scale the degree of this response appropriately.^21^^,^^22^^,^^52^^,^^53^^,^^54^^,^^55^^,^^56^^,^^57^^,^^58^ The CeA also projects back to brainstem taste regions, such as the parabrachial nucleus, and taste-specific responses in these regions in the awake rat are altered by CeA stimulation.^59^ The integration of hedonic and motivational information in the CeA and its efferents to brainstem taste centers and motor centers that control both the jaw and the initiation of movement toward a target of motivation situates the CeA as a limbic command center for consummatory behaviors.^57^^,^^60^ In prior work, opto- or chemogenetic excitation of CeA neurons is often associated with increases in consummatory behaviors,^9^^,^^10^^,^^11^^,^^12^^,^^13^^,^^14^^,^^57^ similar to our findings here. Yet, in our electrophysiological recordings, we noted larger proportions of CeA neurons that showed reductions in firing rate upon port entry and reward consumption, rather than excitations. It is hard to reconcile these findings, but it is possible that optogenetic enhancement of populations normally excited by reward consumption provides the main driver of behavior in these experiments.

Our findings that rats will avidly work for optogenetic activation of the CeA further support the role of CeA in positively motivated behavior and is in line with multiple reports in mice.^11^^,^^22^^,^^27^^,^^28^^,^^50^ That rats will self-stimulate the CeA suggests that the natural activation of at least some of these neurons can participate in a reinforcement process. However, our findings of alcohol- or sucrose-paired enhancement of intake cannot be ascribed to self-stimulation alone, since the amount of intake of solutions paired with stimulation was variable and depended on the specific options available. Importantly, rats typically sampled from both spouts of the available pair of liquids very early in the test session, so the favoring of one reward over another—for example, sucrose over alcohol + laser—is not due to lack of exposure to both rewards for a given offer.

Offers containing alcohol highlight the interaction of preference with optogenetic enhancement of intake. For example, we found that CeA activation during alcohol consumption more than doubled alcohol intake when rats chose between two bottles, each containing some combination of alcohol and/or alcohol with quinine. However, when an alcohol-containing bottle was paired with CeA activation and sucrose was available in the neighboring bottle, the effect was much weaker. Relative to control rats, mean intake of quinine-adulterated alcohol was not increased by optogenetic CeA stimulation when sucrose was also available, and, although the number of lick clusters for alcohol alone was significantly increased when sucrose was in the unstimulated bottle, total intake of alcohol was not significantly altered. Inspection of the values from individual rats clearly shows a large within-group variation on the impact of alcohol-paired CeA activation within the alcohol-sucrose choice. It could be that rats with a greater innate propensity to drink alcohol might be more easily shifted by optogenetic stimulation, yet we did not find a correlation with baseline drinking levels and drinking levels during optogenetic stimulation. Other means to evaluate motivation for alcohol could reveal systematic behavioral underpinnings of the variation we observed. For example, some innate liability to aversion-resistant drinking that is not captured by overall levels of alcohol intake may contribute. It is possible then that individual variation and experience-dependent alterations in the coding of taste of rewards and their resultant value may dynamically influence the role of the CeA in choice. It is possible that requiring a commitment to choice of one option over the other, as opposed to the manner used here where each was freely available and no appreciable effort to shift between options was needed, will aid in understanding the variability arising in these tests where the value of the options was similar. In addition, although no obvious patterns emerged, individual differences in virus expression within the extent of the CeA, or even within different cell types within the CeA, could also account for these distinctions and should be better evaluated within alcohol choice drinking models.

The consistent enhancement of consumption for the most preferred option we observed in alcohol and natural reward pairs is in contrast to a recent report that CeA stimulation can result in the choice of less-preferred cocaine over sucrose.^12^ While the reasons for this difference are unclear, it is important to note that sucrose requires oral consumption, a behavior that we show here engages phasic neural activity attuned to the ongoing motor actions required for continued consumption, whereas cocaine is typically delivered intravenously. Here, we time-locked CeA stimulation to the consumption of the rewards themselves, thereby isolating the behavioral epoch wherein this activation altered intake, in a setting that minimized differences in response costs, effort, and manner of reward or drug intake. In prior studies with cocaine, CeA optogenetic stimulation was applied for a constant 8-s time period following cocaine choice, longer than the period of intravenous delivery, regardless of ongoing behavior during stimualation. Although our stimulation was closely linked to ongoing oral consumption, one caveat to our optogenetic approach is that our stimulation was not applied in a manner consistent with our observations of rhythmic lick-entrainment in CeA neurons. It will be of interest in future experiments to assess if altering intrinsic CeA rhythms during consumption can bias behavior as well.^61^

In our choice tests, we made use of rewards of different identities that differed in their sensory qualities (e.g., taste, smell, viscosity) rather than providing choices between varying values of the same reward. It is possible that the filtering of preference we observed may also apply to differing concentrations or amounts of the same reward.^10^ Given that our rewards were different, it is important to consider whether our optogenetic manipulations impacted behavior primarily by altering processing of sensory features of the tested rewards. We think this explanation is less likely because optogenetic excitation or inhibition during consumption of a reward of a given identity did not always produce the same behavioral effect. Intriguingly, we observed that in two instances CeA stimulation actually drove consumption of the less preferred option when the two rewards available were very similar in value (alcohol vs. alcohol-quinine and sucrose vs. maltodextrin). This could suggest that the function of the CeA is to bias reward preference dependent on the value range of offers available and that alterations in homeostatic state or alcohol dependence could affect this regulation of choice by the CeA.

In agreement with prior work,^21^^,^^58^ our finding that stimulation of the CeA impacted consumption primarily by increasing the number of lick clusters made to the stimulation-paired bottle supports a role for CeA activity in motivation to consume the alcohol and sucrose outcomes. Although we did not find strong evidence of inhibition of the CeA in driving aversion when paired with a preferred tastant, it is possible that there is some aversive quality to CeA inhibition that drives the decrease in consumption when both options were otherwise equal. We cannot discount an influence of CeA neural activity on palatability, although the evidence to support this was weaker, limited to an effect of sucrose-paired or maltodextrin-paired CeA inhibition on the number of licks rats made in each cluster. It was intriguing that these apparent differences in this measure of palatability only occurred when the rats had access to the most valued outcomes. That these changes coincided with overall decreases in total licking at these bottles makes it difficult to totally attribute this loss of consumption to a palatability shift. Intriguingly we observed a greater proportion of lick-modulated neurons in the CeA when sucrose was the outcome available as opposed to alcohol. It is possible that the palatability or motivation to consume sucrose, which is more desirable and more palatable than alcohol, drives this difference in lick-modulation. Future work systematically comparing the activity of individual CeA neurons during choice or consumption of numerous options will be essential for ruling out the potential sources driving this difference in the proportion of lick-modulated CeA neurons we observed. If CeA manipulations can affect outcome palatability, this might suggest that the CeA can separately modify the motivation to consume and the palatability of orally ingested rewards.

The CeA is profoundly impacted by prior experience with drugs of abuse and in particular alcohol. The induction of physical dependence on alcohol in rodents, often via alcohol vapor exposure, recruits the CeA to play an essential role in escalating alcohol drinking, alcohol self-administration, and withdrawal-related anxiety-like behaviors.^62^^,^^63^ These alcohol dependence-induced alterations in CeA circuitry have suggested a limited contribution of the CeA to alcohol-seeking and taking before an individual has met a threshold of drug consumption or is made dependent.^1^^,^^64^ In our experiments, rats volitionally drank alcohol in the homecage, which does not typically induce dependence, yet we found that stimulation of the CeA could promote the consumption of bitter quinine-adulterated alcohol despite the availability of a more preferred non-adulterated alcohol. This, along with the inability of CeA stimulation to flip preference for alcohol if sucrose was available, suggests a broader role for CeA circuits in evaluating currently available rewards and directing motivation to the most desirable option prior to the manifestation of physical dependence.^11^^,^^65^ Moreover, despite low overall levels of alcohol consumption, inhibition of the CeA reduced consumption of inhibition-paired alcohol, similar to what has been reported for cocaine choice.^13^ The ability of the options available to alter the effects of CeA activation and inhibition indicates that the CeA is involved in the relative comparison of value or preference among options. This ultimately provides a potential mechanism for experience and dependence to impinge upon CeA neural processing to promote maladaptive and inappropriate alcohol-seeking and drinking. It will be important in the future to understand how alcohol-induced alterations of CeA might impact the control of alcohol intake we have demonstrated here.

Collectively, our findings suggest the CeA is a critical component of decision-making circuitry that interacts with motivation, preference, and experience to guide the pursuit and consumption of rewards.

### Limitations of the study

The CeA is comprised of a number of diverse cell types which we did not account for in these studies that likely contribute to the enhancement of motivation to seek alcohol and other rewards. For instance, CeA neurotensin-expressing neurons that project to the parabrachial nucleus can control alcohol intake,^11^ and prior studies using natural rewards have implicated prepronociceptin-, somatostatin-, and 5-HT2aR-positive CeA neurons in the promotion of food intake more generally.^21^^,^^22^ Acute and chronic alcohol consumption alters CeA GABAergic and glutamatergic signaling, as well as expression of multiple neuropeptides such as CRF and NPY.^14^^,^^29^^,^^30^ Also, there are important differences in the precise subnuclei of the CeA in promoting diverse behavioral functions that we were not able to capture here, although the previously noted work on neurotensin-expressing neurons in mice points to a role for the lateral CeA.^11^ It will be critical, then, to understand how these diverse populations of cells in the CeA interact to promote alcohol-seeking in a dynamic environment where individuals have an array of desirable options available. Additionally, our studies only included male rats but it will be important in the future to dissect potential sex differences in the contribution of the CeA to alcohol choice.^66^

## STAR★Methods

### Key resources table

**Table:** 

REAGENT or RESOURCE	SOURCE	IDENTIFIER
**Antibodies**		

Mouse anti-GFP	Invitrogen	A-11120; RRID:AB_221568
Alexa Fluor 488 donkey anti-mouse	Invitrogen	A-21202; RRID:AB_141607

**Bacterial and virus strains**		

AAV5-hsyn-ChR2-eYFP	Addgene	26973
AAV5-hsyn-eNpHR3.0-eYFP	Addgene	26972
AAV5-hsyn-GFP	Addgene	50465

**Experimental models: Organisms/strains**		

Long-evans rat	ENVIGO	HsdBlu:LE

**Software and algorithms**		

MATLAB	MathWorks	
GraphPad Prism	GraphPad	

### Resource availability

#### Lead contact

Further information and request for resources should be directed to and will be fulfilled by the lead contact, Patricia Janak (patricia.janak@jhu.edu).

#### Materials availability

This study did not generate new unique reagents.

#### Data and code availability

Data reported in this paper will be shared by the lead contact upon request.

No unique code was generated, but analysis code is available from the lead contact upon request.

Any additional information required to reanalyze the data reported in this paper is available from the lead contact upon request.

### Experimental model and study participant details

Male Long-Evans rats weighing 250–275 g and approximately 60 days of age upon arrival were obtained from ENVIGO (Frederick, MD; *n* = 66) or were bred in our laboratory (*n* = 5). Rats were single-housed in a temperature- and climate-controlled vivarium on a 12-h light:dark cycle. Rats were left undisturbed for at least one week in the vivarium before the beginning of behavioral training, alcohol exposure, or surgery. Water and food was available *ad libitum* and rats were provided with paper shredding enrichment in the homecage. Experimental procedures took place during the light phase of the light:dark cycle. All procedures were conducted in accordance with protocols approved by the Animal Care and Use Committee at Johns Hopkins University.

### Method details

#### Reward solutions

Ethanol was prepared fresh from 200 proof stock solution and diluted in tap water to either 15% by volume for homecage exposure or 10% by volume for electrophysiology and optogenetic experiments. Sucrose (Thermo Fisher Scientific) and maltodextrin (SolCarb, Solace Nutrition) were prepared as 14.2% solutions in tap water by weight. Quinine-adulterated ethanol was prepared by adding quinine salt to a solution of 10% ethanol to achieve a concentration of 100 μM.

#### Surgical procedures

Rats were induced into a surgical plane of anesthesia by inhalation of 5% isoflurane and then maintained at 2–3% isoflurane for the duration of the surgical procedures. For rats in the electrophysiology experiments (*n* = 12) a 1 mm craniotomy was made unilaterally above the central amygdala (AP: −2.4; ML: −4.2 relative to bregma) and 6–8 screws were placed in the skull for anchoring of the implant and one was selected as the screw for the ground wire. A custom-printed microdrive containing a bundle of 16 50 μm tungsten wires and 2 silver ground wires was then lowered slowly to the central amygdala (DV: −7.8 relative to bregma), the ground wires were wrapped around a skull screw, and the drive was secured to the skull with dental cement. For optogenetic experiments, rats received infusions of 500 nL of AAV5-hsyn-ChR2-eYFP (*n* = 22; Addgene 26973; 1.7 × 10^13^ viral particles per mL), AAV5-hsyn-eNpHR3.0-eYFP (*n* = 12; Addgene 26972; 1.0 × 10^13^ viral particles per mL), or AAV5-hsyn-GFP (*n* = 20; Addgene 50465; 1.2 × 10^13^ viral particles per mL) bilaterally into the central amygdala (AP: −2.4; ML: ±4.0; DV: −7.8 relative to bregma) at a rate of 100 nL/min through a 31-gauge gastight Hamilton syringe attached to a Micro4 Ultra Microsyringe Pump 3 (World Precision Instruments) with a 10 min waiting period prior to the removal of the needle. Rats then received with 300 μm diameter optic fiber bilateral implants aimed 0.3 mm above the site of virus infusion (DV: −7.5). Optic fiber implants were secured to the skull with dental cement and 4 skull screws. Rats received an injection of carprofen (5 mg/kg s.c.) immediately following surgery and were allowed to recover for at least 10 days.

#### Histology

Rats were deeply anesthetized with sodium pentobarbitol. For rats with electrode implants, final electrode sites were marked by briefly passing a DC current through each electrode. All rats were then perfused with 4% paraformaldehyde and brains extracted and post-fixed for 24 h at 4C. Brains were cryoprotected in 30% sucrose in 0.1M NaPB for 2–3 days, sliced on a freezing cryostat (Leica), and 50 μm sections were collected. Electrode locations were visualized by staining with cresyl violet. The locations of optical fiber tips and virus expression were visualized with immunohistochemistry. Briefly, slices were washed in 0.1M PBS and blocked in 10% normal donkey serum in 0.1M PBS for 30 min and then incubated at 4C overnight with primary antibody (mouse anti-GFP at 1:1500; Invitrogen A1120). The following day sections were washed in PBS and then incubated for 2 h at RT in secondary antibody (Alexa Fluor 488 donkey anti-mouse at 1:200; Invitrogen A21202) following which they were washed, mounted onto slides, stained with DAPI (Vectashield; VWR H-150) and imaged on a fluorescence microscope (Zeiss).

#### Homecage ethanol exposure

Rats were allowed to drink 15% ethanol freely in the homecage for 24 h Monday, Wednesday, and Friday for either 4 weeks or 5 weeks depending on the experiment. Rats had free access to water the entire time via a Lixit spout in the homecage. Ethanol bottles were weighed before and after each drinking session and rat weights were recorded at the end of each drinking session.

#### Ethanol and sucrose self-administration

For rats in the electrophysiological experiment, a modified self-administration protocol was used. A dish in a recessed port in a modified MedAssociates chamber was filled at the start of the session with 10% ethanol or 14.2% sucrose. During each 40-min session, a 2 s cumulative presence in the reward-containing port resulted in the activation of a pump for 2 s. Based on pilot experiments we determined this matched the rate at which rats consumed the reward and resulted in the fluid dish almost always containing reward (∼0.1 mL per delivery). Licks were recorded from the reward-containing fluid dish via a custom-made lickometer, and port entries and exits were detected by an infrared beam in the recessed port.

#### Electrophysiological recordings

For electrophysiological recordings, rats were tethered via a cable from their headstage to a commutator in the center of the chamber ceiling. Electrical signals and drinking events were collected using the OmniPlex system (Plexon). We recorded from the same location for two sessions if new neurons appeared on previously unrecorded channels. If multiple sessions for the same location were included in the analysis, the same channel was never included more than once. After the second recording in the same location, the drive was advanced 160 μm and recording resumed in the new location at minimum two days later to ensure settling of the tissue around the wires.

#### Two bottle choice with optogenetic manipulation

For the optogenetic experiments, rats were habituated to being tethered to 200 μm core diameter patch cords (Doric Instruments) connected to a commutator (Doric Instruments) in turn connected to a 473 nm DPSS laser (Opto-Engine LLC). During testing rats were placed in a modified home cage that allowed the presentation of two individual bottles via ports on one wall of the homecage with the bottles hanging outside the cage. In daily 30-min sessions, rats were presented with two possible solutions and allowed to freely drink. Licks made on each bottle were recorded using a custom-built lickometer system using Arduino and a capacitive MPR121 sensor (Adafruit Industries). The Arduino recorded licks in real time from each bottle, and one bottle each day was set as the active bottle such that the first lick made to that bottle each second would trigger a TTL pulse to a Master9 Stimulus Controller (AMPI) that dictated the duration and parameters of laser stimulation. For optoexcitation experiments, 473 nm light was delivered for 1s at 20 Hz (5 ms ON, 50 ms OFF) and light output was calibrated to 8–12 mW from the end of the patchcord. For optoinhibition experiments, 532 nm light was delivered continuously from the start of a lick bout until no lick was detected for 1s and light output was calibrated to 15–20 mW from the end of the patchcord. The order of testing, the side of the active bottle and the identity of solutions was counterbalanced across rats. The weight of each bottle was recorded before and after each session and the rats were weighed before each session to identify the amount of solution consumed.

#### Optogenetic intracranial self stimulation

Intracranial self-stimulation was conducted both in the two-bottle choice apparatus described above and in a standard MedAssociates operant chamber. For the two-bottle choice ICSS tests, rats were presented with either empty bottles or bottles containing water for a 30-min session. One bottle, side counterbalanced across tests and rats, was designated as active such that the first lick on that bottle each second triggered a 1s, 20 Hz (5 ms ON, 50 ms OFF) train of 473 nm light bilaterally into the central amygdala with light output set at 8–12 mW from the end of the patchcord. Responses were recorded on the active and inactive bottle as well as the number of stimulations earned. For nosepoke ICSS, rats were placed into a MedAssociates operant chamber, connected to 473 nm lasers via patchcords and commutators and in a 1-h session allowed to nosepoke in either of two ports. One port was designated as active where the first poke in that port each second delivered a 1 s, 20 Hz (5 ms ON, 50 ms OFF) train of 473 nm light bilaterally into the central amygdala with light output set at 8–12 mW from the end of the patchcord. Pokes into each port and stimulations earned were recorded by MedAssociates software.

#### Blood alcohol concentration

Rats were allowed to freely drink 10% alcohol for 40 min in either the operant chamber or in a modified homecage as described above. Immediately after the rat was lightly restrained and blood was collected from a razor nick in the tail into a 70 μL heparanized capillary tube. Blood was then spun down in a centrifuge and plasma was pipetted off and collected. Plasma was then injected and alcohol content analyzed relative to a standard reference using an Analox alcohol content analyzer (AM1, Analox Instruments, Stourbridge, UK).

### Quantification and statistical analysis

#### Electrophysiology data analysis

Isolation of individual units was performed using Offline Sorter (Plexon) by first manually selecting units based on clustering of waveforms. Units were then separated and refined using interspike interval distribution, cross-correlograms, and autocorrelograms. Any units that were not detectable for the entire session were not included in the study. Sorted units were exported to NeuroExplorer 3.0 (Nex Technologies) and MATLAB (Mathworks) for all subsequent analysis. Neurons were determined to be modulated by an event if the spike rate in a custom window (−0.5 to 0.5 s for port entries and port exits and 0 to 0.03 s for lick) following each event significantly differed from a 10 s baseline period according to a Wilcoxon signed-rank test (*p* < 0.05, two-tailed). Peri-stimulus time histograms (PSTHs) were constructed around event-related responses using 0.01 ms bins. The spiking activity of each neuron across these bins of the PSTH was smoothed using a half-normal filter (σ = 6.6) that used activity in previous, but not upcoming, bins. To visualize the normalized activity of neurons, the mean activity within each of the smoothed bins of the PSTH was transformed to a *Z* score as follows: (F_i_ – F_mean_)/F_SD_, where F_i_ is the firing rate of the ith bin of the PSTH, and F_mean_ and F_SD_ are the mean and SD of the firing rate of the 10 s baseline period. Color-coded maps and average traces of individual neurons’ activity were constructed based on these z-scores.

#### Lick-modulation analysis

This analysis was restricted to licks emitted in bouts, i.e., with inter-lick intervals <210 ms. Distributions of spike phases (in radians) were computed for each neuron (neurons with less than 50 spikes in lick cycles were excluded) and non-uniformity was tested with Rayleigh test. Neurons with *p* < 0.01 were considered lick-modulated. V test was used to test for non-uniformity of preferred firing phase distribution with a mean direction of 90° (Figures 2G and S2F). The distributions of the preferred firing phases of lick-modulated neurons from ethanol and sucrose consuming rats were compared using the Kuiper test (circular analog of the Kolmogorov-Smirnov test; Supplement 2G). Proportions of lick-modulated neurons in ethanol and sucrose consuming rats were compared using a z binomial proportion test (Supplement 2H).

#### Statistics

Data are presented as mean ± s.e.m. unless otherwise indicated in the text. Statistical analyses were performed using either MATLAB (Mathworks) or in Prism 8 (GraphPad). For electrophysiological data, statistical tests were performed on unsmoothed data. The specific tests performed are noted throughout the text and figure legends. For electrophysiological data we did not test for normality but made use of nonparametric tests (two-sided Wilcoxon’s rank-sum and signed-rank tests). For optogenetic data we made use of two-way repeated measures ANOVA and post hoc tests were performed with Sidak’s method when appropriate and t-tests performed with Welch’s correction for unequal standard deviations between groups. For the inhibition experiment we had an *a priori* hypothesis to conduct post hoc comparisons within each virus group across the two bottles based on our findings with optogenetic stimulation. Each optogenetic test was conducted only once per rat. Three eNpHR rats did not drink alcohol in any of the tests despite repeated efforts so their data was excluded in these cases, they still performed in all other experiments and were included in those tests.
