# Supplementary material for: Encoding and context-dependent control of reward consumption within the central nucleus of the amygdala
Source: iScience. 2024 Apr 1;27(5):109652. doi: 10.1016/j.isci.2024.109652 (PMC11033178; doi:10.1016/j.isci.2024.109652)
Supplement: Document S1. Figures S1–S6 [file mmc1.pdf]

iScience, Volume *27*

## Supplemental information

# Encoding and context-dependent control of reward consumption within the central nucleus of the amygdala

**Kurt M. Fraser, Tabitha H. Kim, Matilde Castro, Céline Drieu, Yasmin Padovan-Hernandez, Bridget Chen, Fiona Pat, David J. Ottenheimer, and Patricia H. Janak**

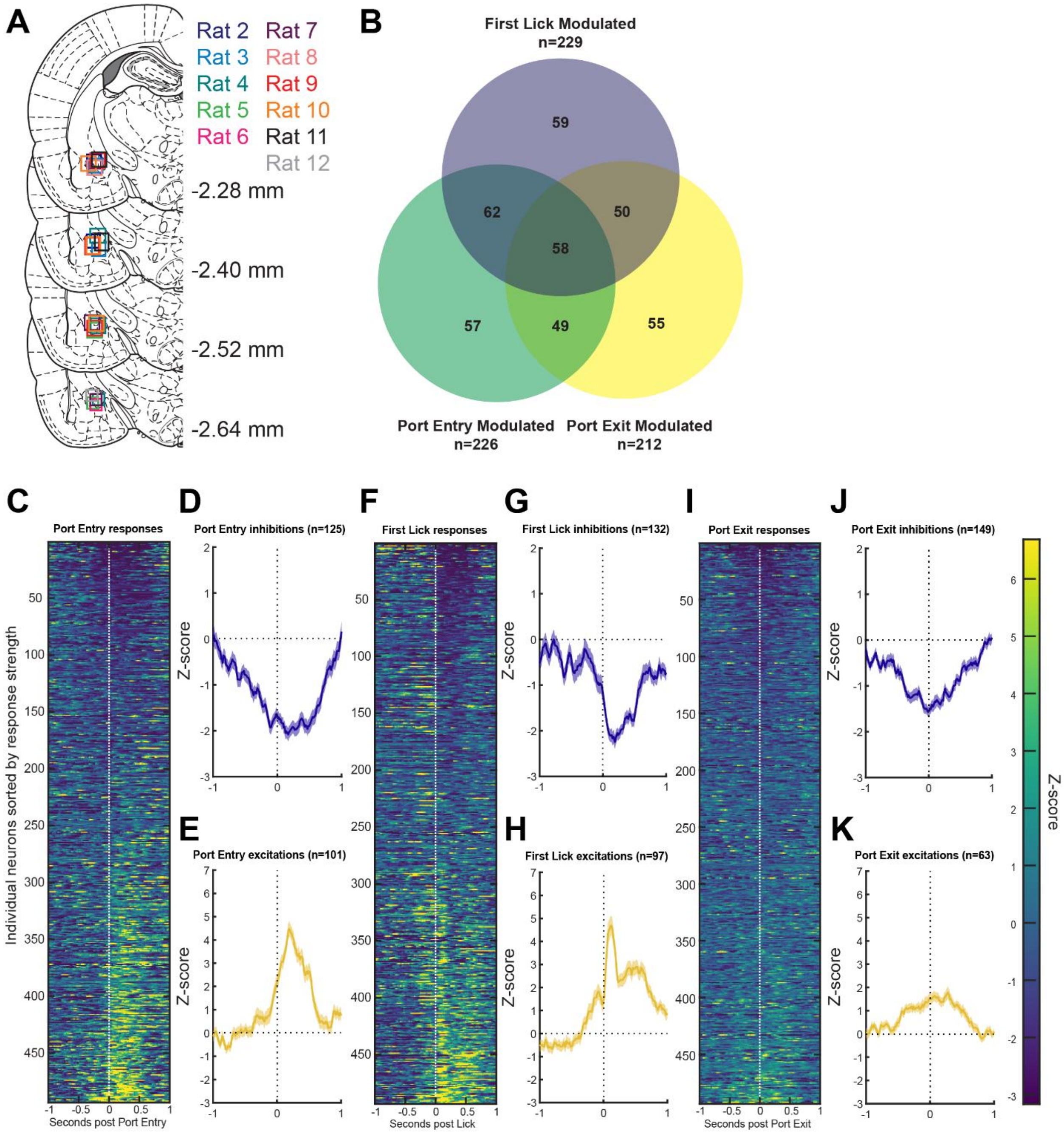


**Figure S1.** Identifying correlates of sucrose consumption in the central nucleus of the amygdala, Related to Figure 1. A) Recreation of recording sites from each of the five rats. The task was identical to that in Figure 1. Rats made on average 50.91 ± 6.637 port entries and 1486 ± 212.4 licks B) Proportion of neurons significantly excited or inhibited by task-relevant events. There were no differences in the proportion of neurons excited or inhibited by port entries ($\chi^2$ = 2.549, p = 0.1104), but more neurons were inhibited than excited by port exits ($\chi^2$ = 34.887, p = 0.0001) and the first lick after a port entry ($\chi^2$ = 5.349, p = 0.0207). In addition, more neurons were inhibited by the the first lick than port exit ($\chi^2$ = 8.7837, p = 0.0124). C) Heatmap of z-scored responses for each neuron recorded sorted by the strength of excitation to port entry. D) Average z-scored response of all neurons that were identified as being significantly inhibited around port entry. E) Average z-scored response of all neurons that were identified as significantly excited around port entry. F-H) Same as C-E but for the first lick post port entry. I-K) Same

as C-E but for port exit. Traces indicate mean z-scored response with overlaid bands indicating ± 1 standard error of the mean.

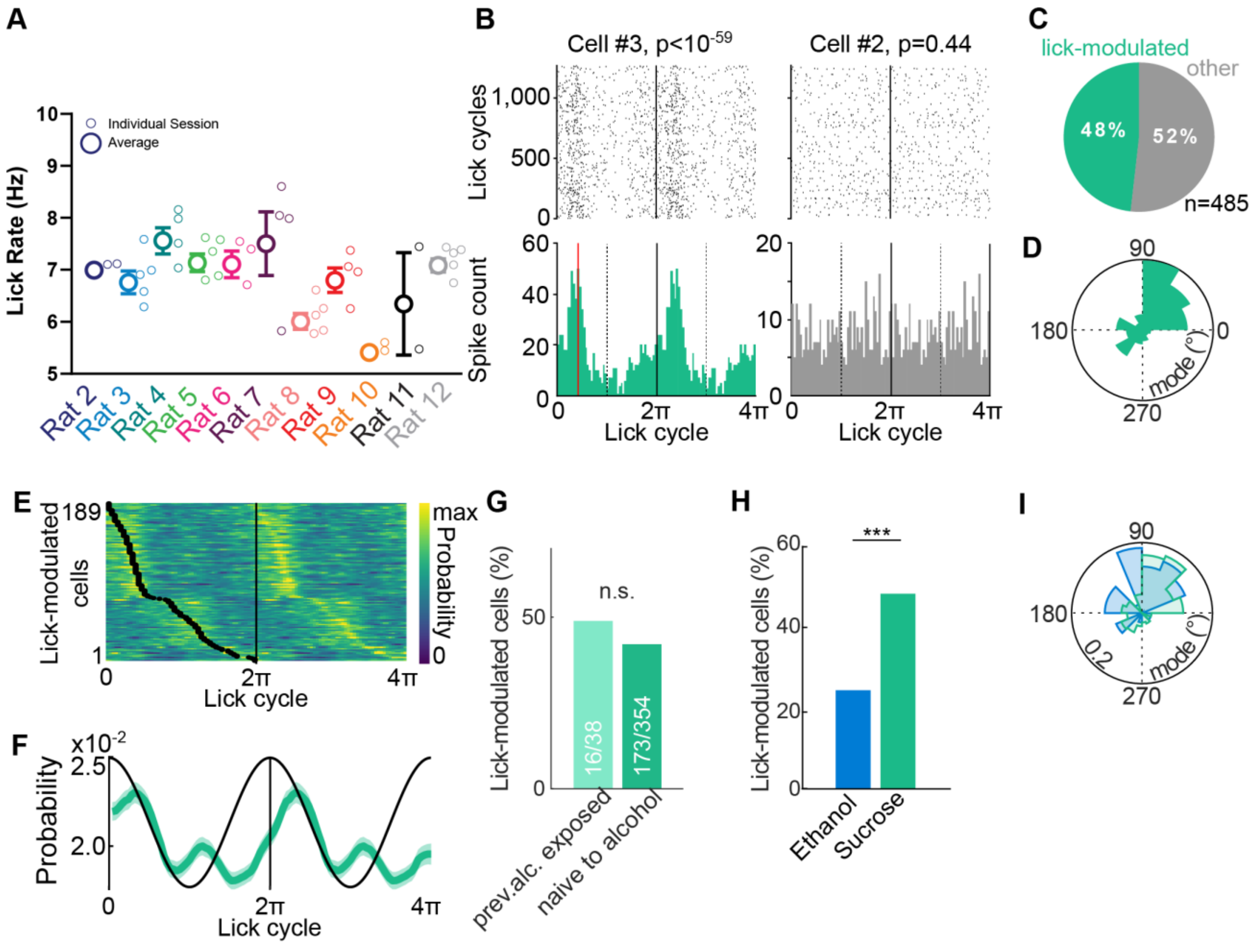


**Figures S2.** Central amygdala neurons are modulated by licks during the consumption of sucrose, Related to Figure 2. A) Average lick rates during the consumption of 14.2% sucrose for each rat during recording sessions. Smaller symbols indicate lick rate for each individual session. B) Spike rasters (top) and histograms (bottom) during lick cycles of two example neurons recorded in the same session. A lick cycle is defined as the time between two consecutive contacts with the fluid delivery port (see Methods). The p-value of Rayleigh test is indicated. C) Proportion of neurons significantly modulated by licks (Rayleigh's test with p-value < 0.01). D) Circular histogram of the preferred firing phases (V test against 90°, n=189 lick-modulated neurons, $V_{189}$ =53.43, $p<10^{-7}$). E) Heat map of spike probability during lick cycles of lick-modulated neurons. Black dots indicate the preferred firing phases (i.e. modes). F) Average spike probability of lick-modulated neurons across lick cycles (mean±s.e.m.). G) There was no difference between the proportion of lick-modulated neurons recorded from rats with (2-5) or without (6-12) a previous history with alcohol (prev. alcohol exposure: 16/38, no prev. alcohol exposure: 173/354 neurons; binomial proportion test, p=0.43). H) The proportion of lick-modulated neurons is higher during sucrose consumption compared to ethanol (z binomial proportion test, $p<10^{-6}$). I) The distributions of the preferred firing phases of lick-modulated neurons from ethanol and sucrose consuming rats are not significantly different (Kuiper test, $k=2.1090.10^{3}$, $K=1.9931.10^{3}$, p=0.1).

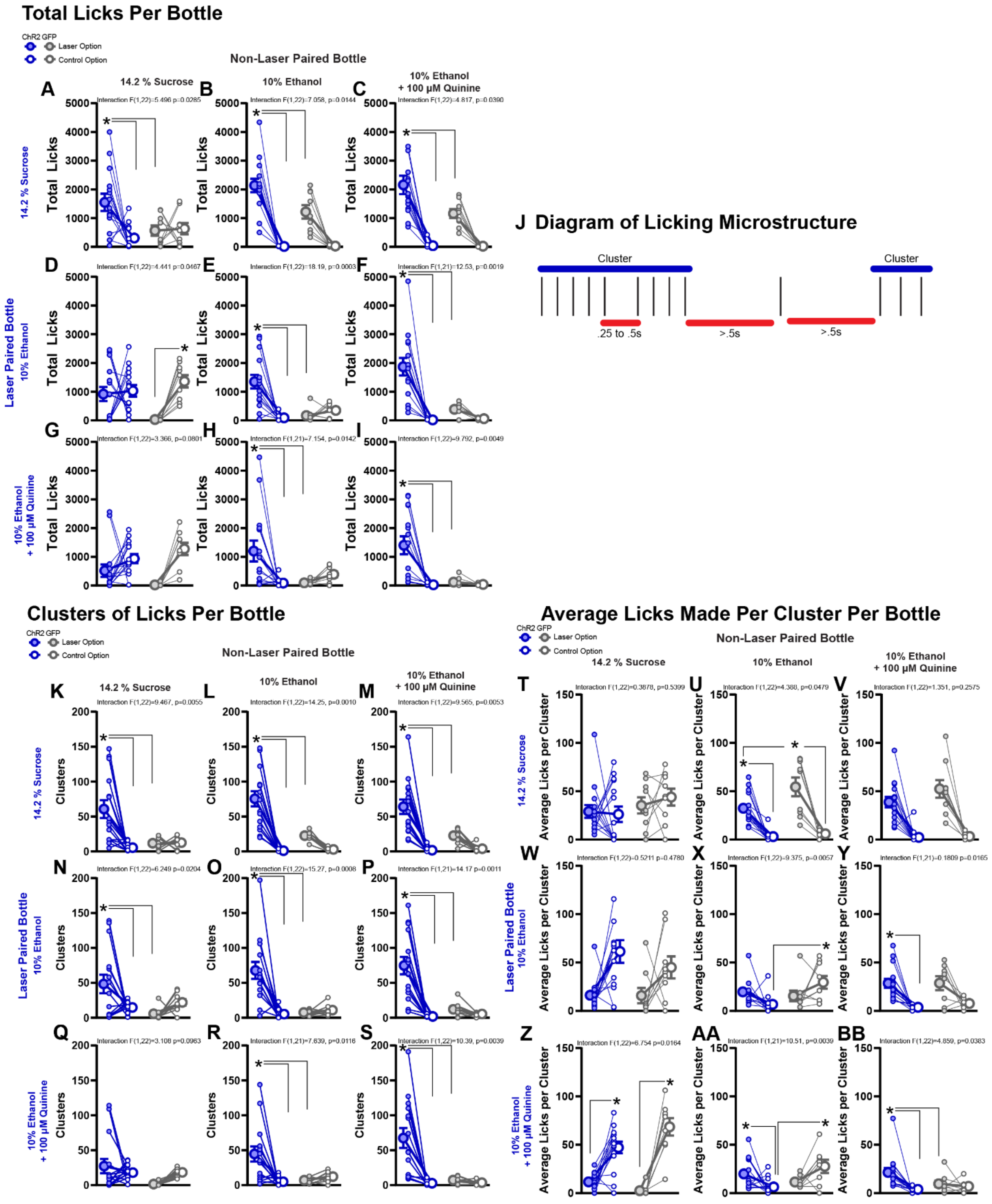


**Figure S3.** Microstructural analysis of consumption indicates optogenetic stimulation of the central amygdala enhances motivation to consume but not palatability of the laser-paired option, Related to Figure 3. A-I) Total number of licks made on each bottle for each of the tests presented in Figure 3. Graphs are organized with the most valued option at the top and leftmost position and the least valued option at the bottom and rightmost position. Comparisons between bottles containing the same offer tile

the diagonal, bottles above diagonal are tests in which the more valued option was blue-light paired and tests below the diagonal are when blue-light was paired with the less valued option J) Diagram of the licking microstructure used to separate out clusters. Clusters had at least three licks and a interlick interval of at least 500 ms. K-S) Same as A-I but the total number of clusters of licks made on each bottle. T-BB) Same as A-I but the average number of licks per cluster made on each bottle. Filled symbols indicate the bottle that resulted in blue light delivery, open symbols the other bottle that did not trigger any light delivery. Large symbols indicate group means ± 1 standard error of the mean and small symbols represent individual rats. * $p<0.05$ for post hoc comparisons made only when an interaction between virus and bottle was observed.

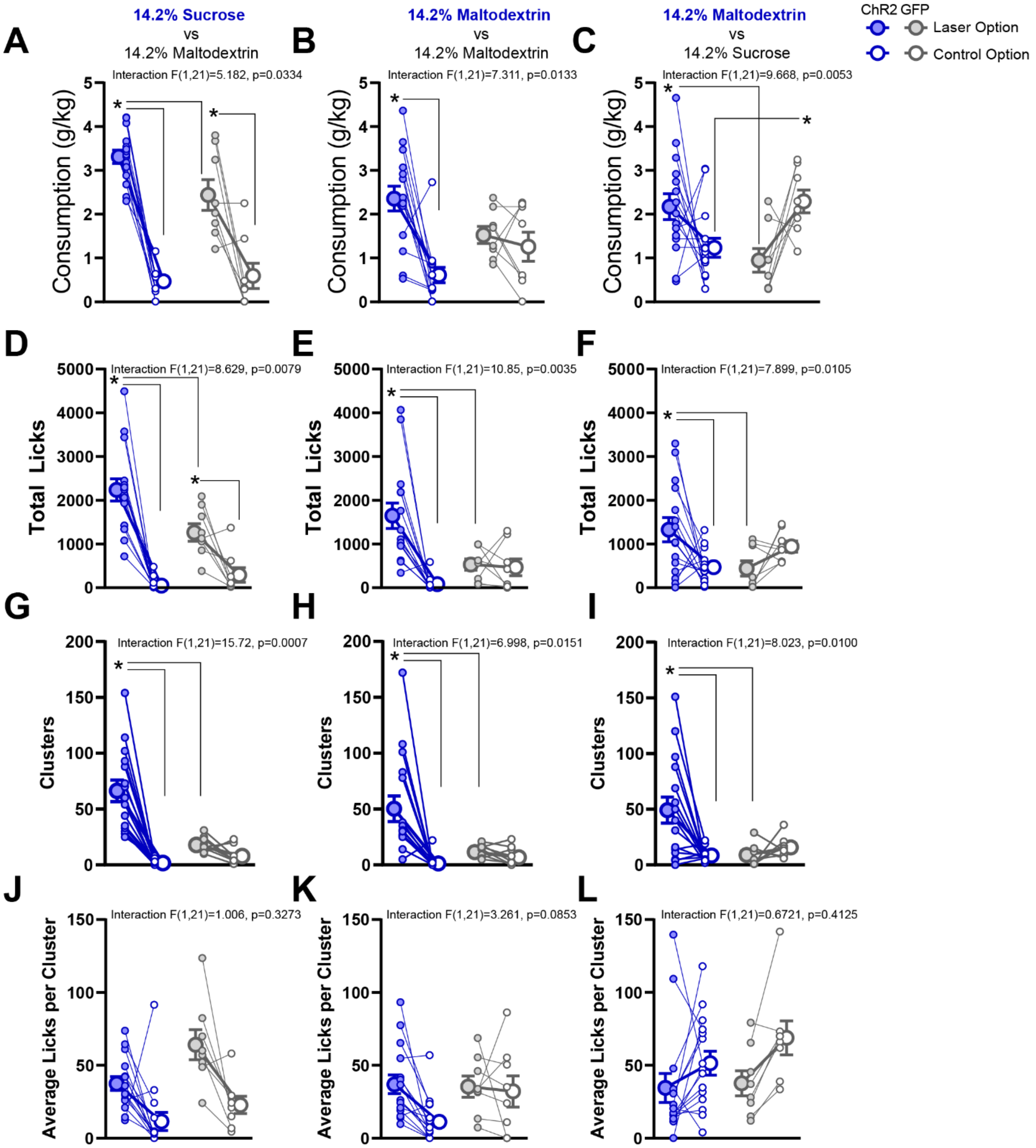


**Figure S4.** Optogenetic stimulation of the central amygdala during consumption can reverse preference between two isocaloric and objectively equal rewards, Related to Figure 3. A) Consumption in g/kg in tests when sucrose consumption was laser-paired and maltodextrin was not. B) Consumption in g/kg in tests where one bottle containing maltodextrin was laser-paired and the other bottle with maltodextrin was not. C) Consumption in g/kg in tests when maltodextrin consumption was laser-paired and sucrose was not. D-F) Same as A-C but the number of licks made on each bottle. G-I) Same as A-C but the number of clusters of licks made on each bottle. J-L) Same as A-C but the average number of licks made per cluster

for stimulation-paired option versus the non-paired option. Filled symbols indicate the bottle that resulted in blue light delivery, open symbols the other bottle that did not trigger any light delivery. Large symbols indicate group means ± 1 standard error of the mean and small symbols represent individual rats. * $p<0.05$ for post hoc comparisons made only when an interaction between virus and bottle was observed.

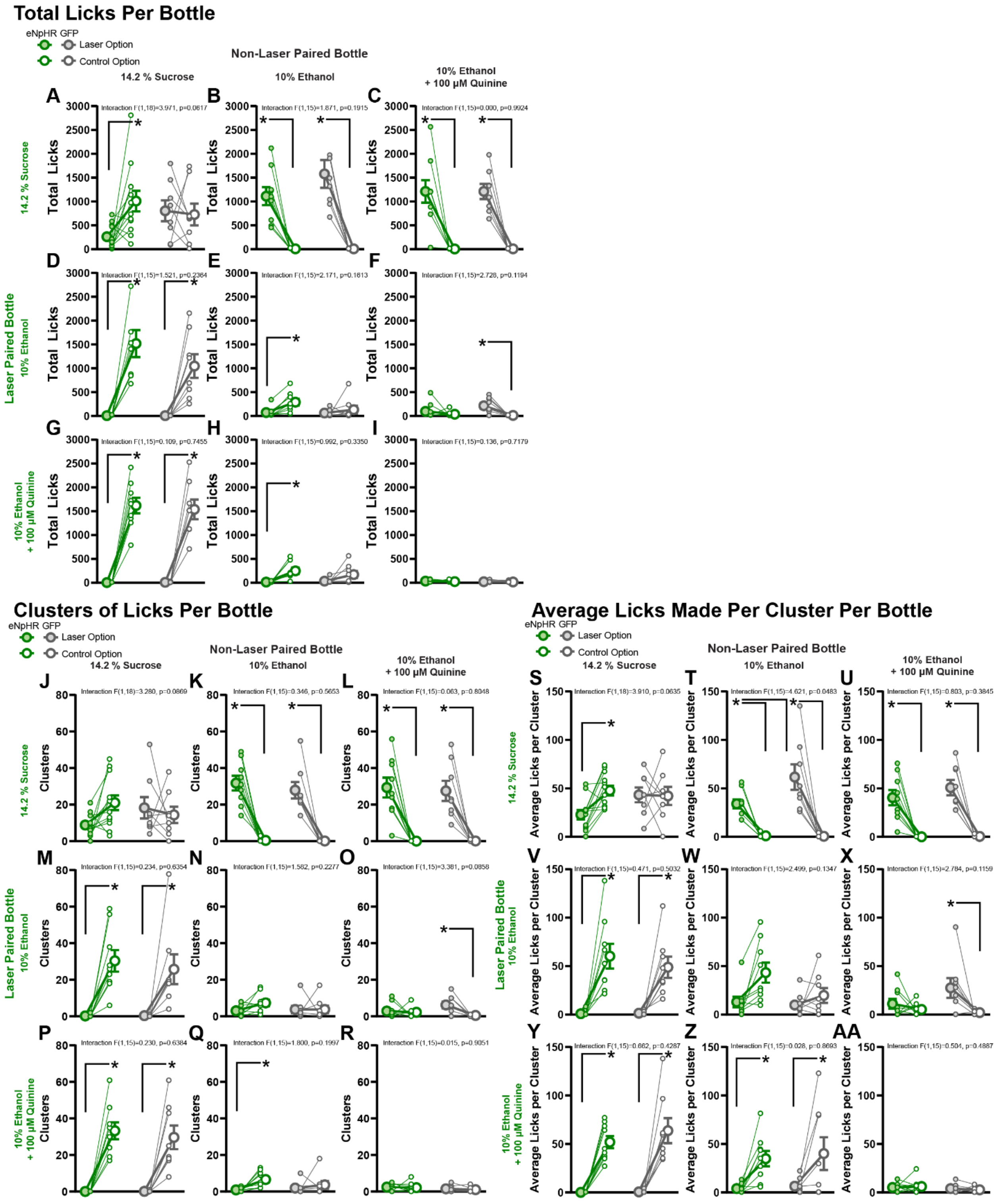


**Figure S5.** Microstructural analysis of consumption indicates optogenetic inhibition of the central amygdala does not suppress motivation to consume but suppresses the palatability of the laser-paired option, Related to Figure 5. A-I) Total number of licks made on each bottle for each of the tests presented in Figure 5. Graphs are organized with the most valued option at the top and leftmost position and the least valued option at the bottom and rightmost position. Comparisons between bottles containing the same offer tile the diagonal, bottles above diagonal are tests in which the more valued option was green-

light paired and tests below the diagonal are when green-light was paired with the less valued option J-R) Same as A-I but the total number of clusters of licks made on each bottle. S-AA) Same as A-I but the average number of licks per cluster made on each bottle. Filled symbols indicate the bottle that resulted in green light delivery, open symbols the other bottle that did not trigger any light delivery. Large symbols indicate group means ± 1 standard error of the mean and small symbols represent individual rats. * p<0.05 for post hoc comparisons.

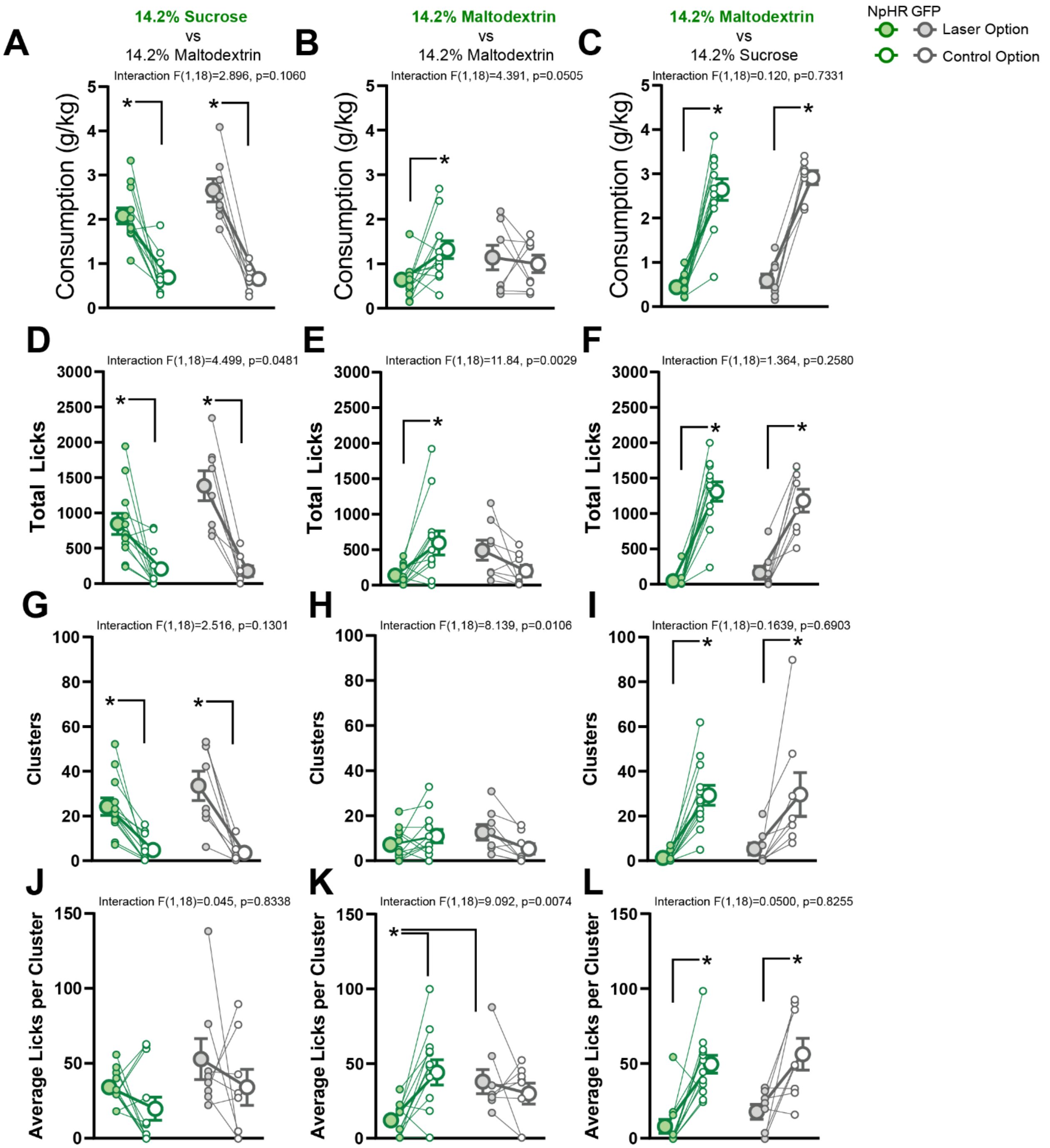


**Figure S6.** Optogenetic inhibition of the central amygdala during consumption cannot reverse preference between two isocaloric and palatable rewards, Related to Figure 5. A) Consumption in g/kg in tests when sucrose consumption was laser-paired and maltodextrin was not. B) Consumption in g/kg in tests where one bottle containing maltodextrin was laser-paired and the other bottle with maltodextrin was not. C) Consumption in g/kg in tests when maltodextrin consumption was laser-paired and sucrose was not. D-F) Same as A-C but the number of licks made on each bottle. G-I) Same as A-C but the number of clusters of licks made on each bottle. J-L) Same as A-C but the average number of licks made per cluster for inhibition-paired option versus the non-paired option. Filled symbols indicate the bottle that resulted in green light delivery, open symbols the other bottle that did not trigger any light delivery. Large symbols

indicate group means ± 1 standard error of the mean and small symbols represent individual rats. * $p<0.05$ for post hoc comparisons.
